# Synergy of BID with doxorubicin in the killing of cancer cells

**DOI:** 10.3892/or.2015.3841

**Published:** 2015-03-09

**Authors:** EMILIA JOANNA ORZECHOWSKA, AGNIESZKA GIRSTUN, KRZYSZTOF STARON, JOANNA TRZCINSKA-DANIELEWICZ

**Affiliations:** Department of Molecular Biology, Institute of Biochemistry, Faculty of Biology, University of Warsaw, 02-096 Warsaw, Poland

**Keywords:** BID, doxorubicin, apoptosis, cell penetrating peptide, HeLa cells, PC3 cells

## Abstract

Overexpression of the BH3-interacting domain death agonist (BID) protein sensitizes certain cancer cell lines to apoptosis induced by anticancer agents, particularly by those acting through death receptors (e.g. TRAIL). Previously, we showed that recombinant BID fused with TAT cell penetrating peptide (TAT-BID) allowed for controlled delivery of BID to different cancer cell lines and moderately sensitized some of them to TRAIL or slightly to camptothecin. In the present study, we showed that TAT-BID delivered to HeLa cells strongly sensitized them to doxorubicin, as identified by cell viability and apoptosis assays. Another cell line sensitized to doxorubicin was PC3, whereas A549 and LNCaP cells were sensitized moderately or not at all, respectively. Sensitization was more pronounced at 1 *μ*M doxorubicin administered for 48 h than for lower doses and shorter treatments. TAT-BID and doxorubicin may thus be considered as a potential therapeutic combination for cervical carcinoma and advanced prostate cancer treatment.

## Introduction

BID (BH3-interacting domain death agonist) protein participates in extrinsic apoptotic signaling (1) and is also able to mediate DNA damage response (2,3). The level of BID is critical for the viability of numerous types of cancer cells since its silencing makes them resistant to apoptosis induced by death receptor ligands (4,5), whereas overexpression sensitizes certain types of cancer cells to TRAIL (6,7) or etoposide (8). Due to the above features, BID has been considered for therapeutic exploitation (4). One of the obstacles associated with its application is the lack of a stringent control of the cellular level of BID, when it is expressed using an adenovirus (7) or pcDNA vectors (3,4), which are commonly used to deliver the protein to cells. As a result, BID could be overexpressed and eventually toxic to cells treated with the vectors. In a previous study, we showed that recombinant BID fused with TAT cell penetrating peptide (TAT-BID) could be delivered to cells in a controlled manner (9). We demonstrated that by using an appropriate dose of TAT-BID it was possible to maintain its concentration at the level which was not toxic alone but sensitized cancer cells to apoptosis, this way increasing the possibility of the potential therapeutic use of the protein. We found that the extent of the effect caused by TAT-BID depended on the cell line used and the anticancer drug employed to induce apoptosis. These results suggested that further studies on the combinations of TAT-BID, an anticancer drug and a cell line might reveal the optimal efficacy of TAT-BID as a potential therapeutic agent. Following that line of thought, in the present study, we examined the sensitization of different cancer cell lines to doxorubicin.

Doxorubicin (DOX; alternative name: Adriamycin) is an anthracycline compound, commonly used as an anticancer drug alone or in combined therapies. Its anticancer molecular action is complex and includes, among others: inhibition of topoisomerases I and II, formation of DNA adducts and free radicals, interaction with membrane proteins, histone eviction and ceramide overproduction (reviewed in refs. 10,11). It has also been shown that administration of DOX alters a transcription profile of the cell (12,13) and that it sensitizes cells to other anticancer drugs (14,15). Due to the broad spectrum of processes affected by DOX, its effects in combined therapies are difficult to predict but rather they need experimental examination. We present here the experimental evidence that TAT-BID strongly sensitized HeLa and PC3 cells to DOX, whereas sensitization of A549 and LNCaP cell lines was moderate or negligible, respectively.

## Materials and methods

Reagents

Doxorubicin (DOX), RPMI-1640 medium, Dulbecco’s modified Eagle’s medium (DMEM), trypsin inhibitor from soybean, fetal bovine serum (FBS), penicillin and streptomycin, D-glucose, sodium pyruvate, MTT reagent and anti-GAPDH antibodies were obtained from Sigma-Aldrich (St. Louis, MO, USA); F12K medium from the American Type Culture Collection (ATCC; Manassas, VA, USA); anti-HA antibodies from Santa Cruz Biotechnology (Dallas, TX, USA); anti-caspase-3 antibodies from Cell Signaling Technology (Danvers, MA, USA); anti-PARP1 and PE-anti-caspase-3 antibodies from Beckton-Dickinson (San Jose, CA, USA); DRAQ5 from BioStatus Ltd. (Shepshed, UK); Dako fluorescent mounting medium from Dako North America (Carpinteria, CA, USA); and Giemsa’s azur and May-Grünwald dyes were from Merck Millipore (Billerica, MA, USA).

### Cell culture

Four cancer cell lines were used in the present study: two prostate cancer cell lines (PC3 and LNCaP) and two non-prostate cancer cell lines: non-small human lung cancer (A549) and cervical carcinoma (HeLa). All cell lines were purchased from the European Collection of Cell Cultures (ECACC). Prostate cancer cells were cultured in RPMI-1640 medium. LNCaP medium contained additionally high D-glucose (4.5 mg/ml), 10 mM HEPES and 1 mM sodium pyruvate. The human non-small lung cancer A549 cell line and cervical carcinoma HeLa cell line were cultured in F12K or DMEM, respectively. All media were supplemented with 10% FBS, penicillin (100 U/ml) and streptomycin (100 *μ*g/ml). Cultures were maintained in a 95% humidified atmosphere of 5% CO_2_ at 37°C. For the experiments, the cells were seeded in 96-well plates, dishes or cover glasses.

### Plasmid construction, mutagenesis, expression, isolation and purification of the recombinant proteins

Plasmids pET28a/TAT-BID encoding BID proteins (wild-type and T59AS76A-mutated variant) fused with the TAT domain were constructed as previously described (9). T59A/S76A variant of TAT-BID was mutated in a manner which made it unphosphorylable by CK2 kinase. Both mentioned proteins were tagged with the His-tag used for purification and with the HA-tag used for simple identification of the protein in the cell. His-tag and the TAT peptide used for the cell penetration were localized at the N-terminal end of the protein; HA tags were placed at the C-terminal end. TAT-BID proteins were expressed, isolated and purified as previously described (9). The protein concentration in the samples used in the experiments was quantified by densitometry after SDS-polyacrylamide gel electrophoresis using ChemiDocXRS (Bio-Rad Laboratories).

### Uptake of the recombinant proteins by the cells

To examine the time-dependent kinetics of uptake and degradation of the recombinant protein by HeLa cells, TAT-BID was added directly to the culture medium at a concentration of 40 *μ*g/ml in the presence of the trypsin inhibitor from soybean (final concentration 0.005%). Whole cell extracts were prepared as previously described (9), and western blot analysis was carried out with the aid of anti-HA antibodies (1:2,000). The membranes were subsequently stripped of the primary antibody and re-probed with anti-GAPDH antibodies (1:100,000). The results were quantified by densitometry using ChemiDocXRS.

### Analysis of cell viability

To examine the effect of doxorubicin (DOX) on cancer cell viability, the PC3, LNCaP, HeLa and A549 cells were seeded in 96-well plates and incubated with different doses of DOX (ranging from 0 to 20 *μ*M) for 24 or 48 h. Similarly, to examine the effect of DOX combined with TAT-BID on cancer cell viability, all tested cell lines were seeded in 96-well plates and treated with 30 *μ*g/ml TAT-BID (for PC3 and LNCaP cells) or 40 *μ*g/ml TAT-BID (for HeLa and A549 cells) alone or in combination with either 0.5 *μ*M or 1 *μ*M DOX for either 24 or 48 h. The same set of experiments were conducted for the wild-type and mutated (T59A/S76A) variant of TAT-BID. Cell viability was analyzed with MTT assay performed as previously described (9).

### Apoptotic assays: procaspase-3 activation and PARP1 cleavage

Procaspase-3 activation and PARP1 cleavage were identified using western blot analysis. HeLa cells were seeded in 60-mm dishes, cultured for 24 h and then treated with 40 *μ*g/ml TAT-BID, 1 *μ*M DOX or both of these agents together for 15 or 18 h, respectively. Next, both floating and adherent cells were collected, washed with PBS, lysed in Laemmli buffer and boiled for 20 min at 95°C. The proteins were then separated on 12% polyacrylamide gel, transferred onto a PVDF membrane, and procaspase-3 (31 kDa) and its active form caspase-3 (large fragment of caspase-3 resulting from cleavage, 17 kDa) were identified using specific anti-caspase-3 antibodies (1:1,000). PARP1 (116 kDa) and its apoptotic fragment (85 kDa) were identified using specific anti-PARP1 antibodies (1:2,000). GAPDH protein was detected as a loading control using anti-GAPDH antibodies (1:100,000). The results were quantified by densitometry using ChemiDocXRS.

### Immunofluorescent staining for active caspase-3

Procaspase-3 activation was also identified using immunofluorescent staining for confocal microscopy observations. HeLa cells were seeded on glass coverslips and cultured for 24 h and then treated with 40 *μ*g/ml TAT-BID, 1 *μ*M DOX or both of these agents together for 15 h. The cells were then washed twice with PBS, fixed with 4% PFA for 10 min at room temperature and washed twice with PBS. Next cells were permeabilized with 0.1% Triton X-100 in PBS for 5 min at room temperature and blocked in 3% BSA in PBS for 3 h at room temperature. After blocking, the cells were incubated with PE-anti-caspase-3 antibodies (1:100 in blocking buffer) for 3 h at room temperature in the dark in a humidified chamber. The cells were then washed twice with 0.05% Tween-20 in PBS, stained with DRAQ (1:1,000 in PBS) and washed again with 0.05% Tween-20 in PBS. Finally the coverslips were mounted on glass microscope slides. Microscopy observations were carried out on a Nikon A1R confocal laser scanning microscope equipped with a Plan Apochromat VC 60x/1.40 oil DIC objective (Nikon Instruments, Melville, NY, USA). For detecting green fluorescence of PE, 488 nm excitation line and 525 nm emission filter were used. For detecting red fluorescence of DRAQ, 641 nm excitation line and 700 nm emission filter were used. Data were analyzed with NIS-Elements imaging software version 4.0 (Nikon Instruments).

### Microscopic observations

To observe morphological changes, the HeLa cells were seeded in 35-mm dishes, cultured for 24 h and then treated with 40 *μ*g/ml TAT-BID, 0.5 *μ*M DOX or both of these agents together for 24 or 48 h, respectively. The cells were then washed twice with PBS and fixed with ice-cold methanol for 10 min at 4°C. Next, the cells were washed twice with PBS and stained with 0.25% May-Grünwald for 3 min at room temperature. After that 0.1 M phosphate buffer pH 7.0 was added (1:1) and left for 5 min. Next, the cells were stained with 0.76% Giemsa’s azur for 15 min at room temperature, washed at least three times and dried. Images (magnification, x100) were captured using a Nikon eclipse TE200 microscope equipped with a Nikon Digital Camera DXM 1200 (Nikon Instruments).

### Data analysis

All experiments carried out as cell viability measurements were repeated at least five times, and for each individual point at least five independent measurements were made. The results are shown as an average ± SD. Western blot analysis and microscopic observations were carried out in triplicate and representative results are presented. Differences between groups were calculated using the Studen’ts t-test. A P-value <0.05 was considered to indicate a statistically significant result. The following ranges were defined: P<0.05, P<0.01 and P<0.001. Statistica version 10 software (StatSoft, Inc., Krakow, Poland) was used for analysis.

## Results

Previously, we found that TAT-BID moderately sensitized PC3 and A459 cells, and slightly sensitized HeLa cells to TRAIL, while it was ineffective in the sensitization of LNCaP cells (9). We also observed slight sensitization of PC3 cells but not the remaining cell lines to camptothecin. Due to the diversified effects of TAT-BID on the above mentioned cell lines as previously observed, we examined in the present study the same cell lines in terms of their sensitization by TAT-BID to DOX.

**Figure 1:**
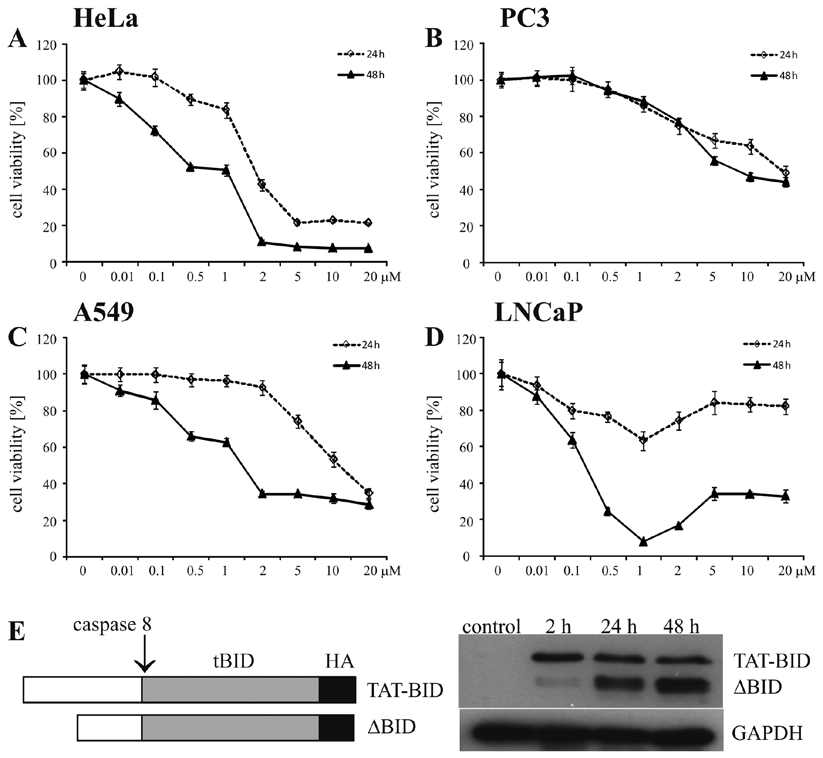
Characteristics of the experimental system used in the present study. (A–D) Viability of cancer cell lines treated with different doses of doxorubicin (DOX) (0–20 *μ*M) for 24 or 48 h as determined by MTT assay. The average cell viability (± SD) is shown. (E) Degradation of TAT-BID taken up by HeLa cells during the treatment period. Protein was identified in the whole cell extracts using antibodies against HA-tag localized at the C-terminal end of the polypeptide. GAPDH was used as a loading control and detected using specific antibodies. The arrow on the scheme indicates a cleavage site for caspase-8 in the undegraded TAT-BID and in the degradation product ΔBID.

The sensitization of cancer cells to TRAIL and camptothecin was previously determined to be optimally visible for concentrations of both drugs that, when administered alone, resulted in a relatively small decrease in cell viability (9). To exploit the same idea in the present study, we tested the effects of DOX acting alone on the viability of particular cell lines. There was a broad range of sensitivity of the examined cell lines to DOX (Fig. 1A–D and Table I). Moreover, different types of sensitivity patterns were observed for different cell lines in terms of their dose- and time-dependence of cell viability (Fig. 1A–D). The decrease in cell viability of the HeLa and A549 cells treated with DOX was both dose- and time-dependent. Sensitivity patterns of prostate cancer cells to DOX were different from those of the HeLa and A549 cell lines. In the case of PC3, there was no time-dependent effect on the viability of PC3 cells treated with DOX; the decrease in viability after a 24-h treatment was similar or the same as after a 48-h treatment (Fig. 1B). In the case of LNCaP cells, the effects of DOX depended on the time of the treatment; however, a simple dose-dependence was not observed. The maximal reduction in cell viability was at 1 *μ*M DOX, followed by weakening of the effect observed for higher concentrations of DOX (Fig. 1D). Such a pattern suggested an additional factor that appeared at higher concentrations of DOX and counteracted the lethal action of DOX. It has been previously shown that treatment of prostate cancer cells with DOX results in the elevated expression of multidrug-resistance proteins (16). It is, thus, possible that such a process occurred in the LNCaP cells and interfered with the lethal action of DOX at concentrations >1 *μ*M.

Based on the above observations, we selected the concentration of 0.5 *μ*M DOX to be used in the further experiments. When this concentration was used for a 24-h treatment, a small or moderate decrease in viability was noted in all cell lines. To be closer to the effective range of DOX, we additionally used 1 *μ*M DOX. Moreover, we also tested a 48-h administration of DOX at both concentrations to observe the effects resulting from a long-term DOX treatment (12–15) on the sensitization by TAT-BID. The latter conditions raised a question of whether TAT-BID remained stable for >24 h to influence the apoptosis induced by DOX after that time. In fact, we observed degradation of TAT-BID inside the cells. The amount of degraded TAT-BID accounted only for 23% of the TAT-BID present in the cells after 2 h but this value increased up to 62% after 24 h and to 75% after 48 h (Fig. 1E). However, the total amount of TAT-BID in the cells increased during the treatment and was 1.5-fold and 2-fold higher after 24 and 48 h, respectively, than after 2 h of the treatment. As a result, an absolute amount of intact TAT-BID in the cells after 48 h decreased by only ~30% as compared to the amount present in the cells after a 2-h treatment. Moreover, the majority of degraded TAT-BID remained in the form of a 26–27 kDa polypeptide (ΔBID) comprising 222–230 C-terminal residues as it was detected by anti-HA antibodies (Fig. 1E). This means that ΔBID included the active tBID fragment and a sequence accessible to caspase-8. Taken together, we conclude that the recombinant active form of BID (tBID) was available and influenced apoptotic signaling during the entire period of the experiment.

**Figure 2:**
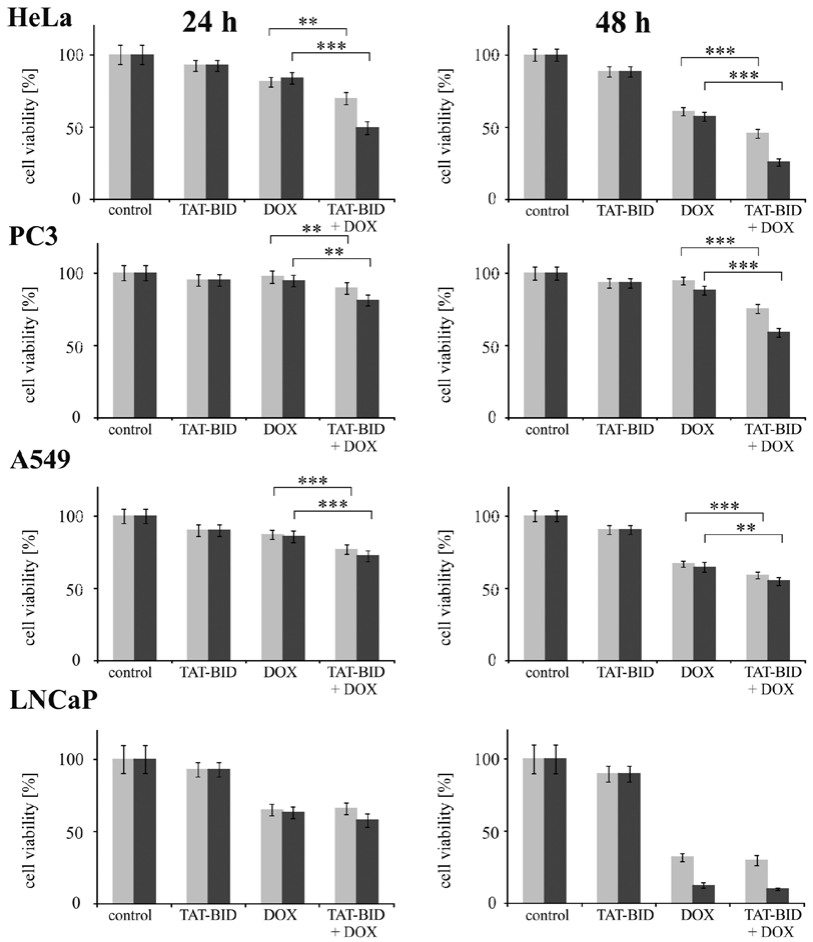
Effect of TAT-BID on the viability of different cancer cell lines treated with doxorubicin (DOX) as determined by MTT assay. Cells were treated with 30 *μ*g/ml TAT-BID (for PC3 and LNCaP cells) or 40 *μ*g/ml TAT-BID (for HeLa or A549 cells) alone or in combination with either 0.5 *μ*M (light bars) or 1 *μ*M DOX (dark bars) for either 24 or 48 h. The average cell viability (± SD) is shown.

TAT-BID sensitized particular cell lines to DOX to different extents (Fig. 2 and Table II). Statistically significant changes were observed for HeLa, PC3 and A549 but not for LNCaP cells (Fig. 2). Among the first group, the highest sensitization was found for HeLa and PC3 cells. This was reflected by a reduction in the cell viability observed when TAT-BID appeared in addition to DOX, which was ~30% for both cell lines treated with 1 *μ*M DOX for 48 h. In the A549 cell line such a reduction was only slightly >10% (Fig. 2). This was also reflected by the synergy between DOX and TAT-BID, calculated here as the coefficient of drug interaction (CDI) (17). A slight synergistic effect (CDI, 0.8–0.9) was observed in the PC3 and HeLa cells treated with 0.5 *μ*M DOX for 48 h. A moderate synergistic effect (CDI, 0.7–0.8) was found for PC3 cells treated with 1 *μ*M DOX for 48 h and a significant synergistic effect (CDI<0.7) for HeLa cells treated with 1 *μ*M DOX for either 24 or 48 h (Table II). In the latter case, the synergistic effect for TAT-BID and DOX was more pronounced than any effect calculated for previously described (9) combinations of TAT-BID with other anticancer agents: TRAIL and camptothecin (Table II).

**Figure 3:**
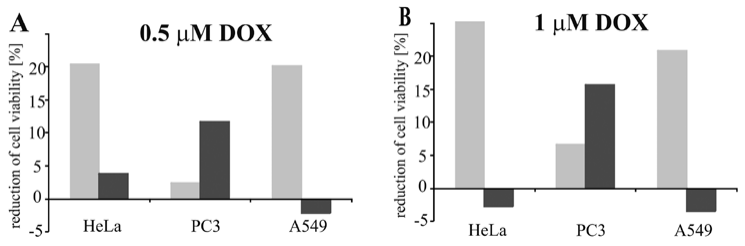
Time-dependent reduction in the viability of cells treated with either doxorubicin (DOX) alone (light bars) or with TAT-BID + DOX combination (dark bars). The reduction in cell viability that occurred between the 24- and 48-h treatment was calculated from data presented in Fig. 2 as follows: [(control) −(DOX treated)]_48 h_ − [(control) − (DOX treated)]_24 h_ for DOX acting alone, and [(DOX treated) − (TAT-BID + DOX treated)]_48 h_ − [(DOX treated) − (TAT-BID + DOX treated)]_24 h_ for TAT-BID + DOX combination. (A) The reduction in cell viability for 0.5 *μ*M DOX. (B) The reduction in cell viability for 1 *μ*M DOX.

A distinct feature of the sensitization of HeLa and A549 cell lines to DOX was that in both cases the extent of sensitization by TAT-BID was poorly dependent on whether the cells were treated for 24 or 48 h. This was illustrated by an additional increased reduction in cell viability observed when DOX was supplemented by TAT-BID which was relatively stable upon prolongation of treatment time. This was in contrast to the reduction in cell viability observed upon addition of DOX alone which was clearly time-dependent (Fig. 3). The reverse pattern was found for PC3 cells. A decrease in cell viability observed upon addition of DOX alone was independent on time, whereas an additional increased reduction in cell viability observed when TAT-BID was combined with DOX was time-dependent (Fig. 3).

We also tested the sensitization of all cell lines by the mutant TAT-BID^T59A/S76A^ that cannot be inactivated by cellular CK2 kinase. It has been shown that phosphorylation of BID at T59 and S76 might protect BID from activation by caspase-8 cleavage and eventually from further processing of the apoptotic signal (18). However, we previously demonstrated that the mutant TAT-BID^T59A/S76A^ is as effective in the sensitization of cells to TRAIL and camptothecin as the wild-type TAT-BID (9). In the present study, we found the same effectiveness of the mutant in the sensitization of all the cell lines to DOX (data not shown).

**Figure 4:**
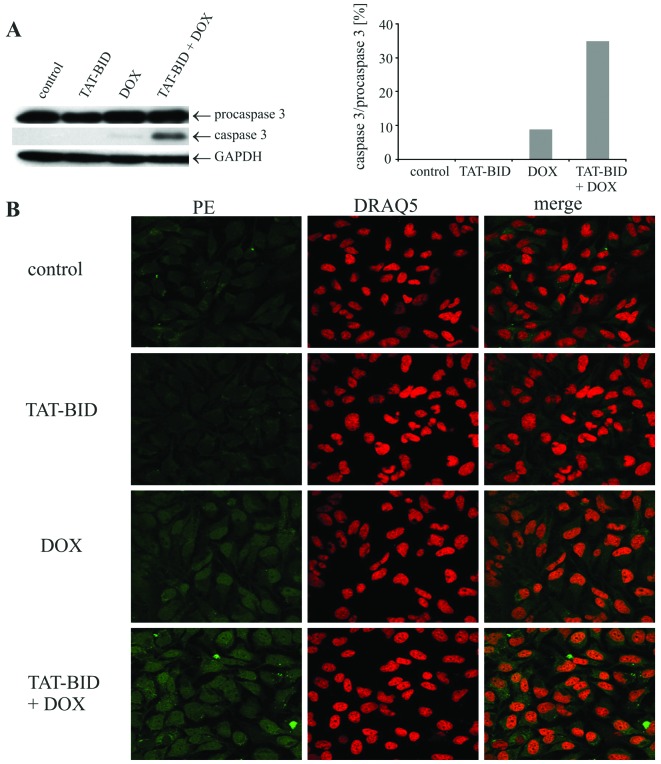
Effect of TAT-BID on the activation of procaspase-3 induced in HeLa cells by doxorubicin (DOX). Cells were treated with 40 *μ*g/ml TAT-BID or 1 *μ*M DOX (or both) for 15 h. (A) Western blot analysis of procaspase-3 (31 kDa) cleavage into the active caspase-3 form (large fragment, 17 kDa). Caspase-3 and GAPDH were identified using specific antibodies in whole cell extracts. (B) Microscopic images of the cells. Cells were stained with PE-anti-caspase-3 antibodies (active caspase-3, green) and DRAQ5 (nuclei, red) and observed using confocal microscopy.

**Figure 5:**
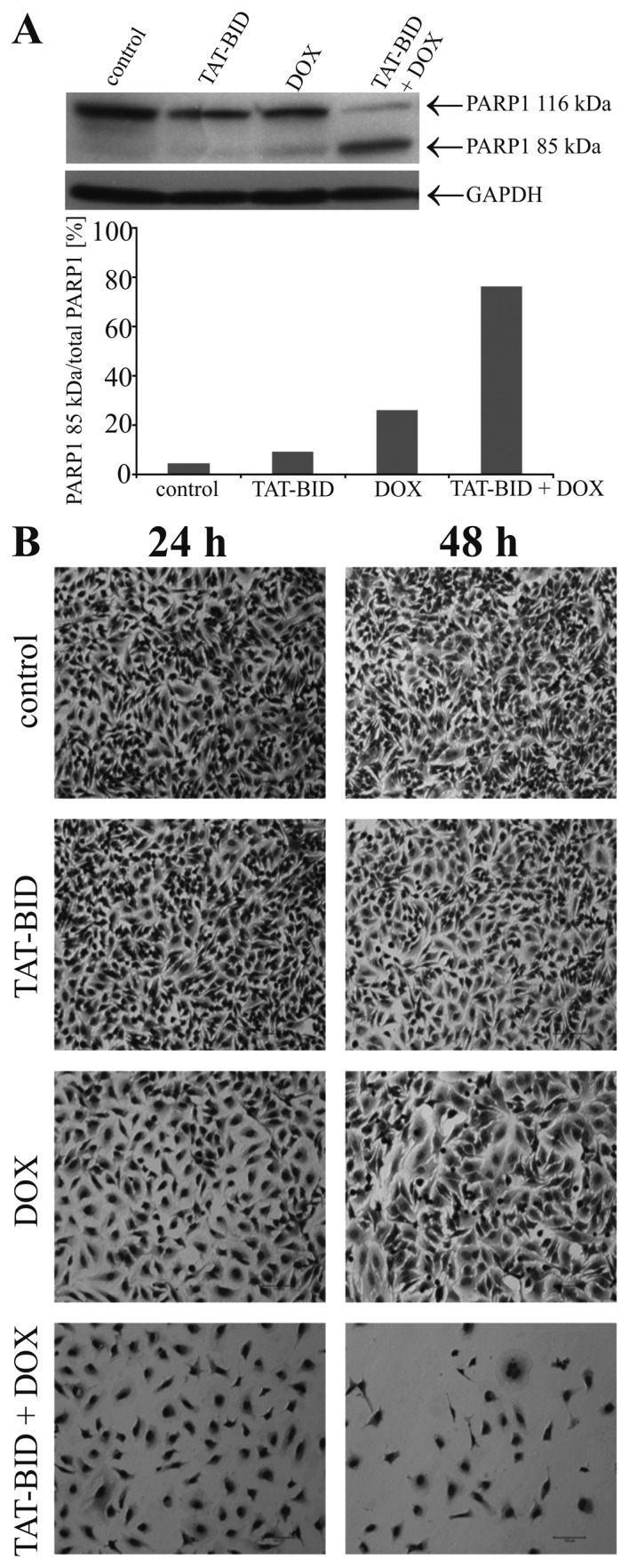
Effect of TAT-BID on progression of apoptosis induced in HeLa cells by doxorubicin (DOX). (A) Effect of TAT-BID on PARP1 cleavage in HeLa cells treated with DOX. Cells were treated with 40 *μ*g/ml TAT-BID or 1 *μ*M DOX (or both) for 18 h. PARP1 (116 kDa), its apoptotic form (85 kDa) and GAPDH were identified in whole cell extracts using specific antibodies. (B) Microscopic images of the cells. Cells were treated with 40 *μ*g/ml TAT-BID or 0.5 *μ*M DOX (or both) for 24 or 48 h. Cells were stained with Giemsa’s azur and May-Grünwald dyes.

To gain a deeper insight into the mode of action of TAT-BID combined with DOX, we performed additional experiments on the HeLa cell line which was chosen since it was effectively sensitized by TAT-BID to DOX (Fig. 2), and since the synergistic effect between TAT-BID and DOX was high for this cell line (Table II). We found that sensitization of the cells as revealed by MTT assay resulted from increased apoptosis achieved upon combined treatment. In the presence of both TAT-BID and DOX, after a 15-h treatment we observed activation of procaspase-3, both by western blot analysis (Fig. 4A) and microscopic observations (Fig. 4B). This was followed by enhanced PARP1 cleavage (18-h treatment; Fig. 5A), and eventually by pronounced changes in cell appearance and a decrease in their number (24- and 48-h treatment; Fig. 5B).

## Discussion

The main finding presented in the present study is that externally delivered recombinant BID was unexpectedly efficient in killing HeLa and PC3 cells when it was combined with DOX. This observation is the first of all relevant findings supporting the potential use of TAT-BID + DOX as a therapeutic combination. In a previous study (9), we found that TAT-BID combined with TRAIL was effective against PC3 and A549 cells, suggested its anticancer potential in the treatment of advanced prostate cancer and non-small human lung cancer. This study showed that cervical carcinoma and prostate cancer cells appear even a better target for TAT-BID when it is combined with DOX.

DOX is widely clinically used in the treatment of different types of cancers. However, toxicity in the brain, liver, kidneys and particularly in the heart are serious issues associated with DOX chemotherapy (10). Therefore, reducing the DOX dosage by combined administration with a synergistically acting factor is an effective therapeutic option (19). The combination of TAT-BID and DOX exhibited the most pronounced synergy at 1 *μ*M DOX (Table II). This was the concentration at which DOX acting alone decreased only weakly or at most moderately the viability of HeLa and PC3 cells, and higher doses of DOX were needed to achieve an effect similar to that of the TAT-BID + DOX combination (compare Figs. 1 and 2). Therefore, TAT-BID may be considered as a factor that potentially reduces the side-effects of DOX owing to a lower drug dosage.

In addition to suggesting the potential therapeutic use of TAT-BID in combination with DOX, this study presents some observations and raises various questions concerning the role of BID in the apoptosis induced by DOX in different types of cancer cells. The main observation is that the extent of sensitization of particular cell lines by TAT-BID is specific for DOX and does not overlap with those observed previously for TRAIL or camptothecin (9). Several different models have been proposed for DOX-mediated cell death (10–15). It has been suggested that the specific pathway to cell death induced by DOX depends on the concentration of the drug, the time of treatment and cancer type (10). This is in agreement with the distinct sensitivities of different cell lines to DOX (Table I), and with the different patterns of dependence of DOX effects on the time of treatment and on the DOX dose (Fig. 1), observed here when DOX was administered alone. This is also in agreement with the specific DOX sensitization of particular cell lines by TAT-BID. The only exception to the latter feature is the lack of sensitization to all examined agents [(9) and the present study] observed for LNCaP cells which most possibly results from impairment in signaling localized upstream from BID (20).

Questions raised by the above observation include: why the effect of TAT-BID was more pronounced in PC3 and HeLa than in A549 cells, and why extra BID delivered to HeLa cells was so efficient in supporting the apoptosis induced by DOX and not by TRAIL or camptothecin.

Concerning the PC3 cells, meaningful synergy was observed only after a 48-h and not after a 24-h treatment (Table II). There may have been a specific time-dependence in the effects caused in PC3 cells by DOX acting alone or in the presence of TAT-BID, distinct from those observed for HeLa and A549 cells. The effects of DOX on PC3 cells were not dependent on time when DOX acted alone but they were when TAT-BID was present (Fig. 3). This pattern suggests that there were finite resources of a critical factor necessary for the progression of DOX-induced apoptosis in the PC3 cells which, however, were exhausted within the first 24 h of the treatment but were still present when TAT-BID was added. It also means that the critical factor was BID protein. Such a sharp dependence of the effect of DOX on the BID level may explain the reason for the pronounced sensitivity of PC3 cells to TAT-BID combined with DOX observed after 48 h. A specific feature of PC3 cells making them distinct from the other cells used here is the lack of active p53 (21). p53 has been shown to be critical for the effectiveness of DOX due to regulation of the accumulation of DOX in the cells (22). We previously linked a lack of active p53 in PC3 cells with higher sensitivity of these cells to TAT-BID combined with camptothecin due to the absence of the p53-dependent apoptotic pathway induced by DNA damage (9). Such a mechanism may also be the reason for the specific time-dependence of DOX effects in PC3 cells observed in the absence and in the presence of TAT-BID, and eventually in pronounced sensitivity to TAT-BID combined with DOX.

In regards to the HeLa cells, no conclusive data can explain its marked sensitization to DOX by TAT-BID. Detailed studies on the mechanism of DOX-induced apoptosis in HeLa cells identified no alteration in expression of basic apoptotic proteins, BID, BAX, Bcl-2, Bcl-xL and p53, during the first 48-h treatment (23). Another report demonstrated a small but significant decrease in Bcl-2 and an increase in the BAD level observed in HeLa cells after a 18-h treatment with DOX (19). Since BAD increases the sensitivity of cells to BID (24), alterations in Bcl-2 and BAD levels after long-term treatment might together contribute to the sensitization of HeLa cells to DOX.

## Figures and Tables

**Table I:** IC_50_ values calculated for different cell lines treated for 48 h with DOX.

Cell line	IC_50_ (*μ*M)
PC3	8.00
A549	1.50
HeLa	1.00
LNCaP	0.25

**Table II:** Coefficients of drug interaction (CDI) for TAT-BID and DOX administered to different cell lines under different conditions.

Cells	DOX 0.5 *μ*M, 24 h	DOX 0.5 *μ*M, 48 h	DOX 1 *μ*M, 24 h	DOX 1 *μ*M, 48 h	TRAIL^a^	CPT^a^
PC3	0.9961	*0.8518*	0.9001	**0.7165**	**0.7663**	*0.8831*
A549	0.9813	0.9719	0.9392	0.9360	**0.7929**	0.9347
HeLa	0.9296	*0.8485*	0.6400	0.5129	*0.8653*	0.9418
LNCaP	0.9719	1.0341	0.9360	0.9102	0.9697	0.9572

a CDI for TAT-BID and TRAIL and TAT-BID and campthotecin (CPT) are shown for comparison (9). Synergy: 0.8–0.9, italics; 0.7–0.8, bold; <0.7, underlined.
